# Supplementary material for: An Immuno-Separated Assay for Ochratoxin Detection Coupled with a Nano-Affinity Cleaning-Up for LC-Confirmation
Source: Foods. 2022 Apr 15;11(8):1155. doi: 10.3390/foods11081155 (PMC9026555; doi:10.3390/foods11081155)
Supplement: Supplementary file 1 [file foods-11-01155-s001.zip › Figure S1.pdf]

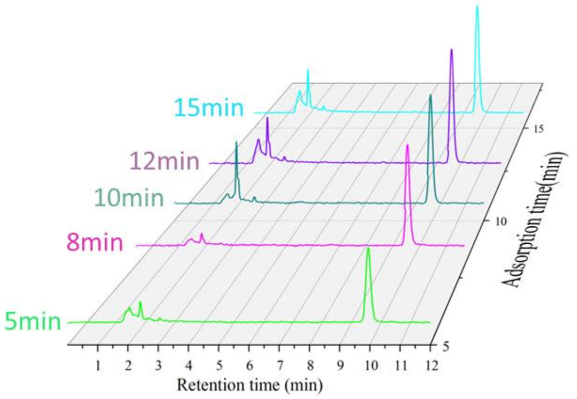

Twenty-ng OTA was diluted in 1.0 mL assay buffer for separation by IMAs with gradient adsorption time 5, 8, 10, 12, and 15 min. .

Each separated IMAs was eluted for LC detection, respectively.

It could be observed that the capture of OTA by IMAs reached balance after 10 mins.

(a) Immuno-separated kinetics of OTA by IMAs

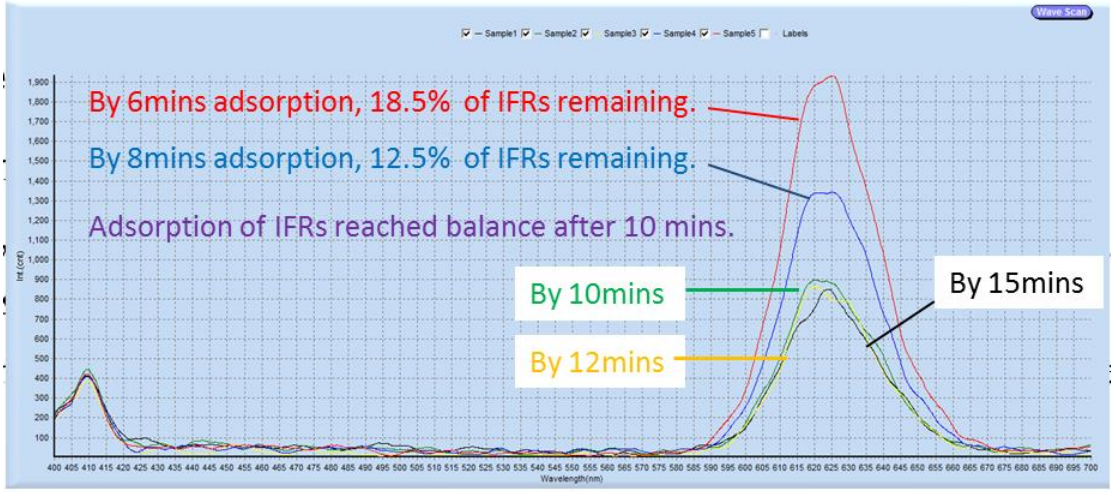

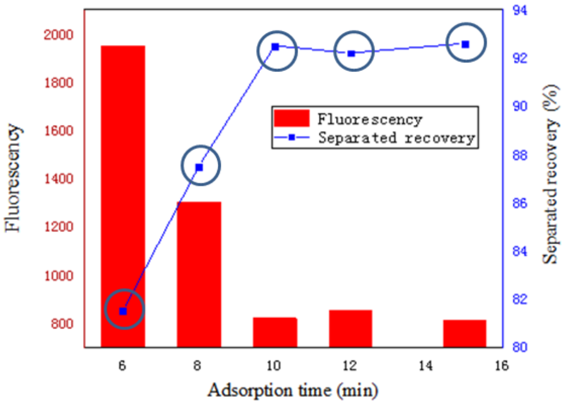

IFRs with level of 0.13μg was diluted in 500μL assay buffer for separation by IMAs with gradient adsorption time of 6, 8, 10, 12, and 15 min.

Each attempt IFRs/IMAs was separated in designed time, then supernatants for fluorescent detection. Results proved that the capture of IFRs by IMAs reached balance after 10 mins.

(b) Immuno-separated kinetics of IFRs by IMAs

Figure S1. Immuno-separated kinetics of OTA and IFRs by IMAs.
