# Supplementary material for: An Immuno-Separated Assay for Ochratoxin Detection Coupled with a Nano-Affinity Cleaning-Up for LC-Confirmation
Source: Foods. 2022 Apr 15;11(8):1155. doi: 10.3390/foods11081155 (PMC9026555; doi:10.3390/foods11081155)
Supplement: Supplementary file 1 [file foods-11-01155-s001.zip › Figure S2.pdf]

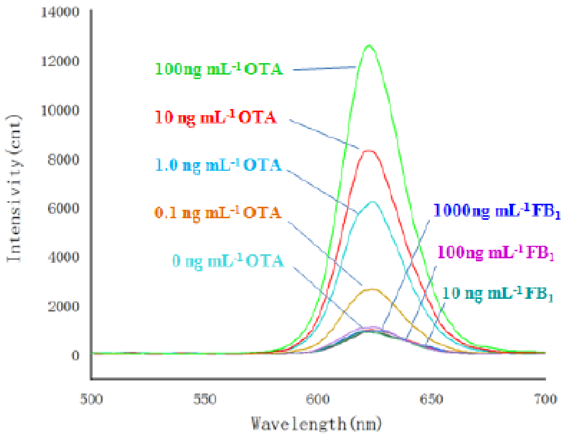

(a)

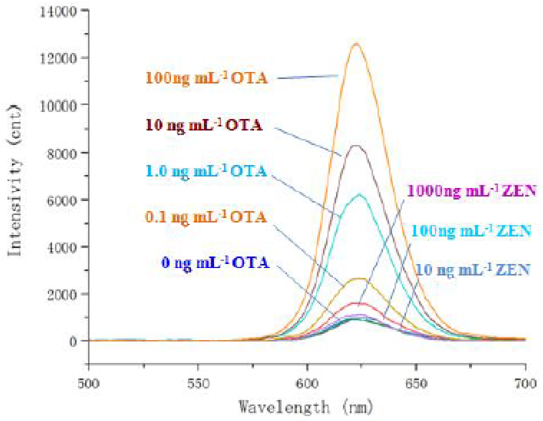

(b)

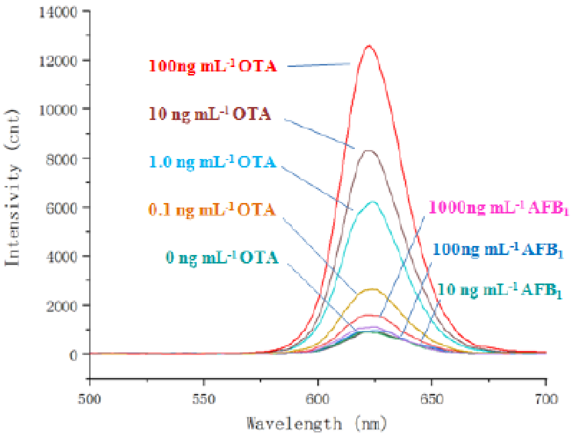

(c)

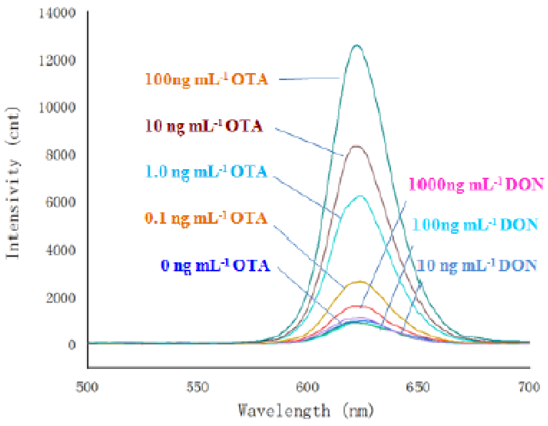

(d)

Figure S2. Cross-reactivity detection of Aflatoxin B_1, Zearalenone, Fumonisin B_1 and Deoxynivalenol. (a) Immuno-separated kinetics of OTA by IMAs. (b) Immuno-separated kinetics of IFRs by IMAs. Figure-S2 Cross-reactivity detection of Aflatoxin B_1, Zearalenone, Fumonisin B_1 and Deoxynivalenol. (a) Cross-reactivity detection of Fumonisin B_1 (FB_1). (b) Cross-reactivity detection of Zearalenone (ZEN). (c) Cross-reactivity detection of Aflatoxin B_1 (AFB_1). (d) Cross-reactivity detection of Deoxynivalenol (DON).
