# Supplementary material for: An Immuno-Separated Assay for Ochratoxin Detection Coupled with a Nano-Affinity Cleaning-Up for LC-Confirmation
Source: Foods. 2022 Apr 15;11(8):1155. doi: 10.3390/foods11081155 (PMC9026555; doi:10.3390/foods11081155)
Supplement: Supplementary file 1 [file foods-11-01155-s001.zip › Figure S3.pdf]

1. Detection of rice sample by immuno-separated assay

1.1. Sample collection

Rice is the main food crop in South China. Detection of mycotoxin residues in rice is a major task to ensure food safety.

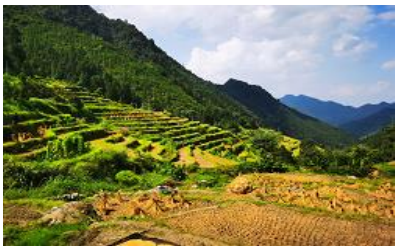

Grain samples were collected from rice fields just harvested last month in a famous terraced village in Shaoguan City.

1.2. Sample pretreatment

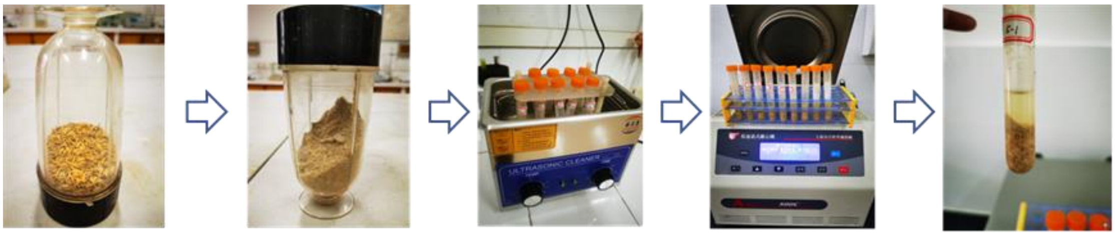

(1) Take 100g of rice and grind it with a crusher.

(2) Two gram of OTA-spiked rice sample was extracted with 4.0mL of extraction buffer by ultrasonic for 15 min.

(3) The sample was centrifuged at 9000g for 10min. 400μL of supernatant was ready for the immuno-separated assay.

Note: 400μL of supernatant was equal to 0.2g of rice sample.

1.3. Sample detection

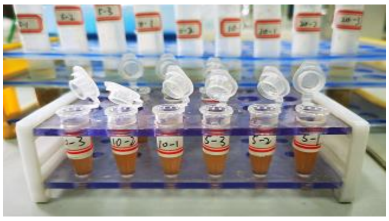

(1) 400μL supernatant and 800μL assay buffer were mixed with 3μg of IMAs, then shaking for 15mins.

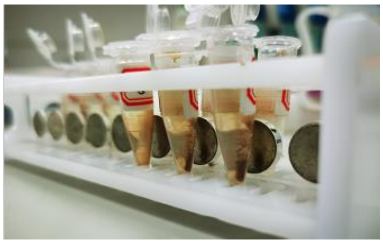

(2) OTA was collected by immuno-separation. The volume of the sample solution has no effect.

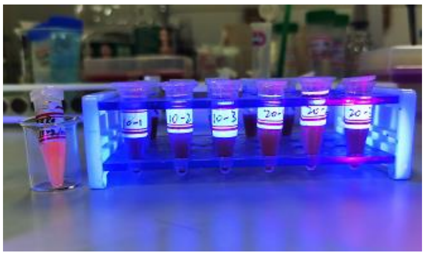

(3) IFRs were mixed with IMAs to evaluate the OTA adsorption level. This process for standards and samples must be in parallel.

1.4. Detection results

The immuno-separatied assay is as same as other immunoassays that detection of OTA for standard curve shall be carried out at the same time as the detection of samples.

The worldwide MRLs of OTA for most commodities ranges from 5 to 20 ppb. As to spiked OTA levels of 5-20ppb, six points of standards (0, 1, 3, 10, 30, and 100 ppb) are enough for the detection of spiked rice samples.

(1) Standard curve

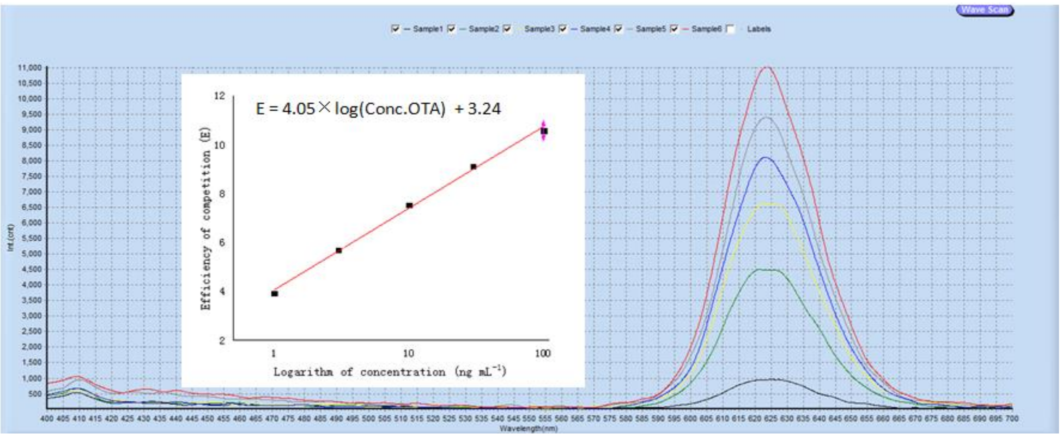

(2) Results of detection of 5.0ng mL^{-1} spiking rice samples

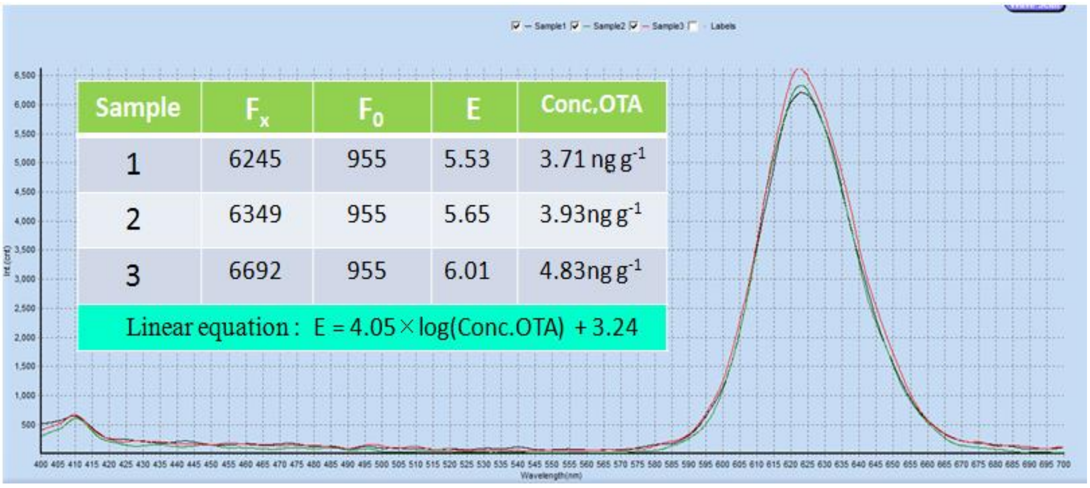

(3) Results of detection of 10ng mL^{-1} spiking rice samples

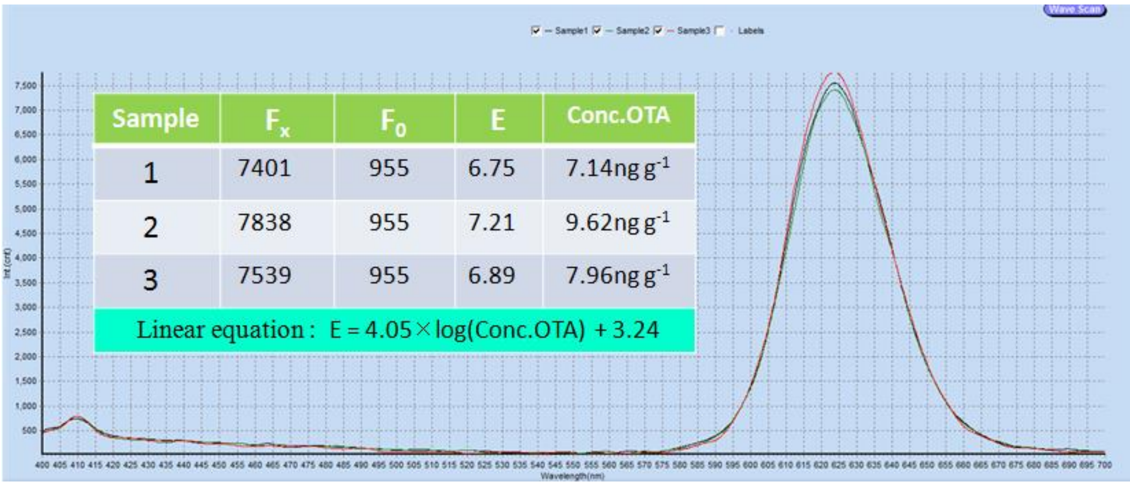

(4) Results of detection of 20ng mL^{-1} spiking rice samples

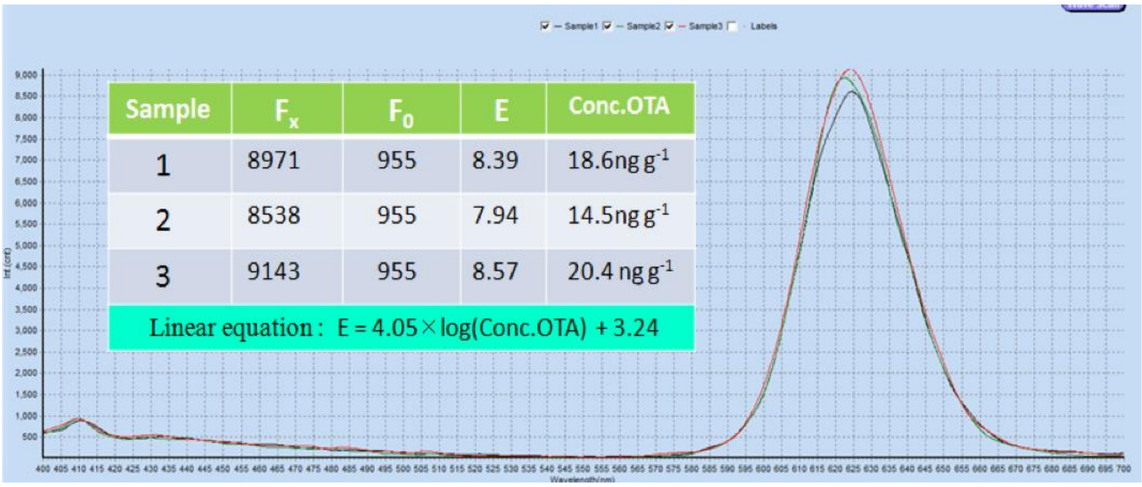

2. Detection of beer sample by immuno-separated assay

2.1. Sample pretreatment

(1) Beer samples were treated with ultrasonic to drive away the CO_2 gas.

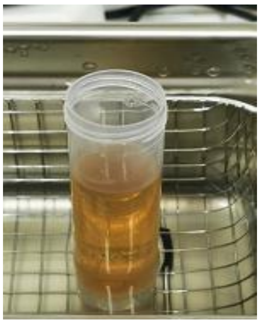

(2) 2.0mL spiked samples were diluted by 2.0mL extraction buffer following ultrasonic treatment to avoid adsorption of OTA by matrix of beer.

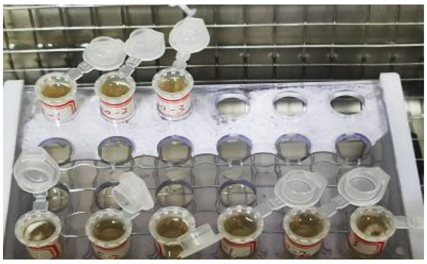

2.2. Sample detection

(1) 400μL treated samples and 800μL assay buffer were mixed with 3μg of IMAs, then shaking for 10mins.

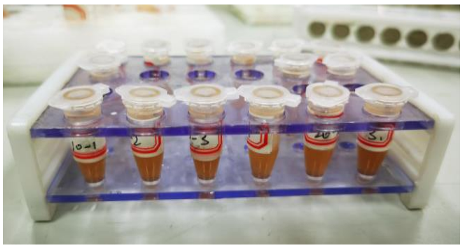

(2) OTA was collected by immuno-separation. The volume of the sample solution has no effect.

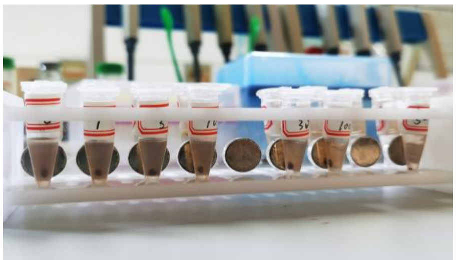

(3) IFRs were mixed with IMAs to evaluate the OTA adsorption level. This process for standards and samples must be in parallel.

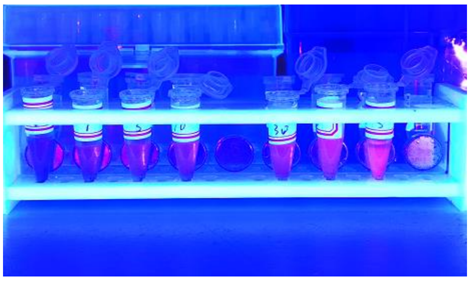

2.3. Detection results

(1) Standard curve

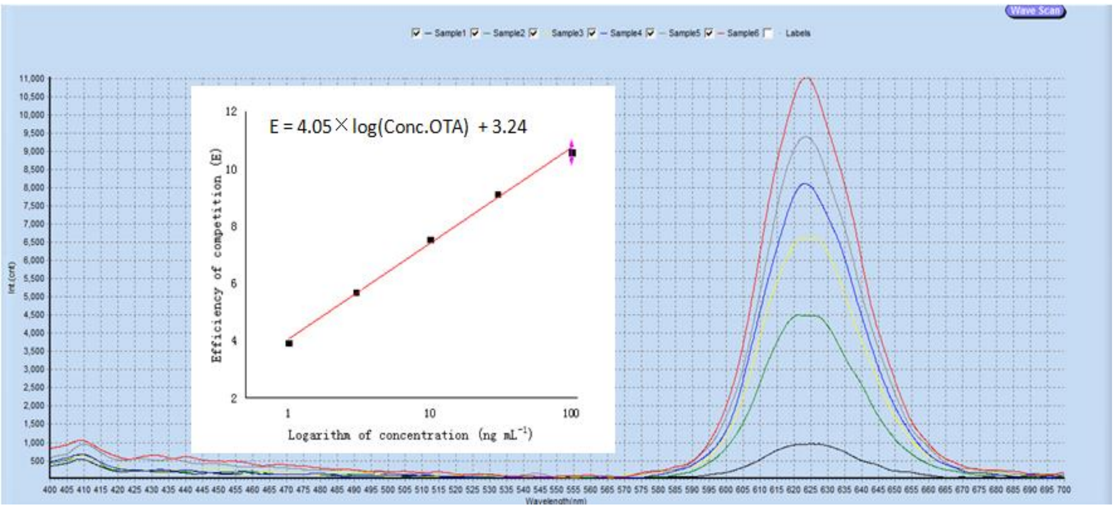

(2) Results of detection of 5.0ng mL^{-1} spiking beer samples

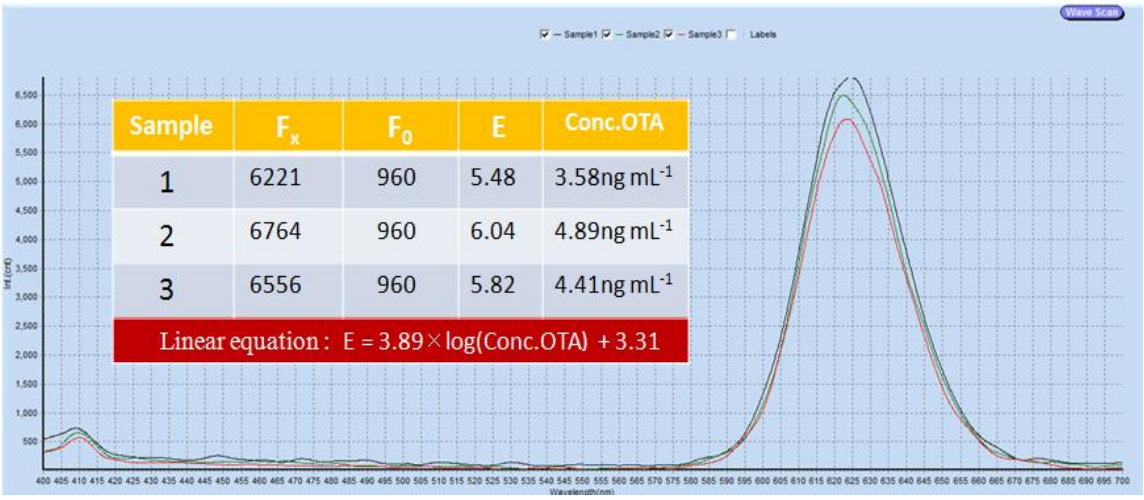

Sample	F_x	F_0	E	Conc.OTA
1	6221	960	5.48	3.58ng mL^{-1}
2	6764	960	6.04	4.89ng mL^{-1}
3	6556	960	5.82	4.41ng mL^{-1}
Linear equation： E = 3.89×log(Conc.OTA) +3.31				

(3) Results of detection of 10ng mL^{-1} spiking beer samples

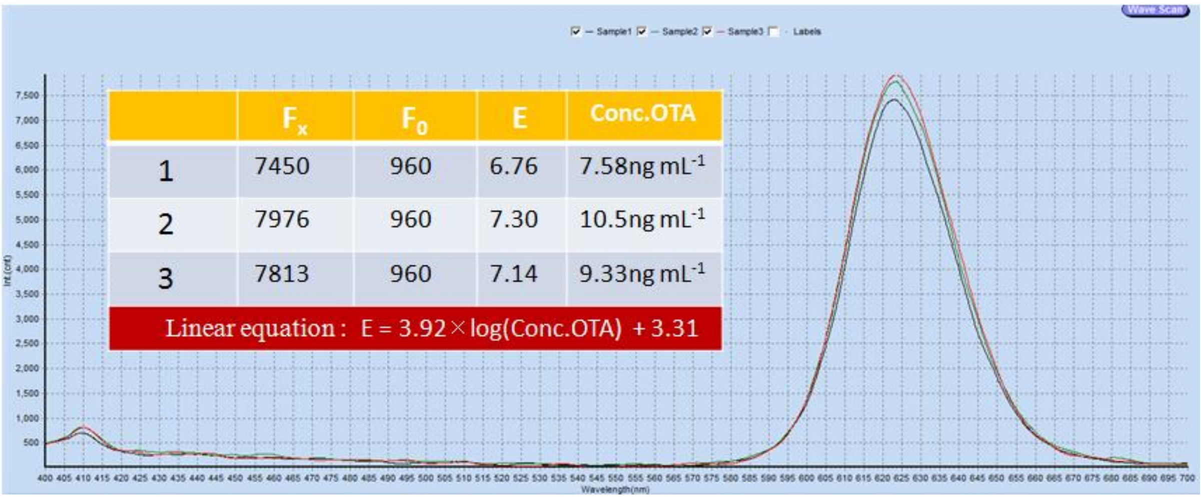

	F_x	F_0	E	Conc.OTA
1	7450	960	6.76	7.58ng mL^{-1}
2	7976	960	7.30	10.5ng mL^{-1}
3	7813	960	7.14	9.33ng mL^{-1}
Linear equation : $E = 3.92 \times \log(\text{Conc.OTA}) + 3.31$				

(4) Results of detection of 20ng mL^{-1} spiking beer samples

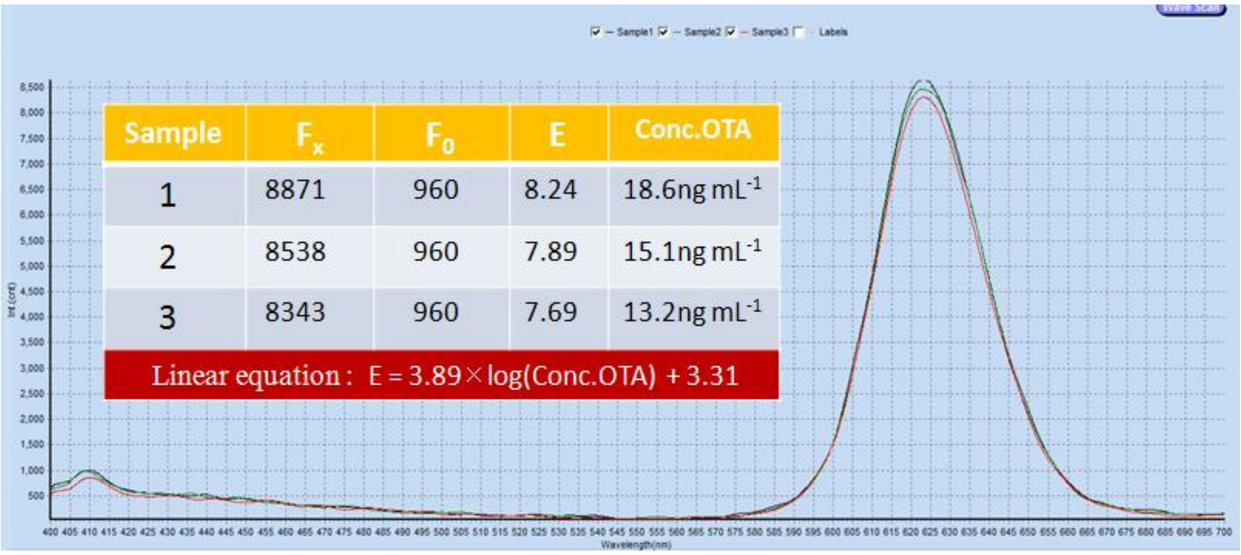

Sample	F_x	F_0	E	Conc.OTA
1	8871	960	8.24	18.6ng mL^{-1}
2	8538	960	7.89	15.1ng mL^{-1}
3	8343	960	7.69	13.2ng mL^{-1}
Linear equation : $E = 3.89 \times \log(\text{Conc.OTA}) + 3.31$				

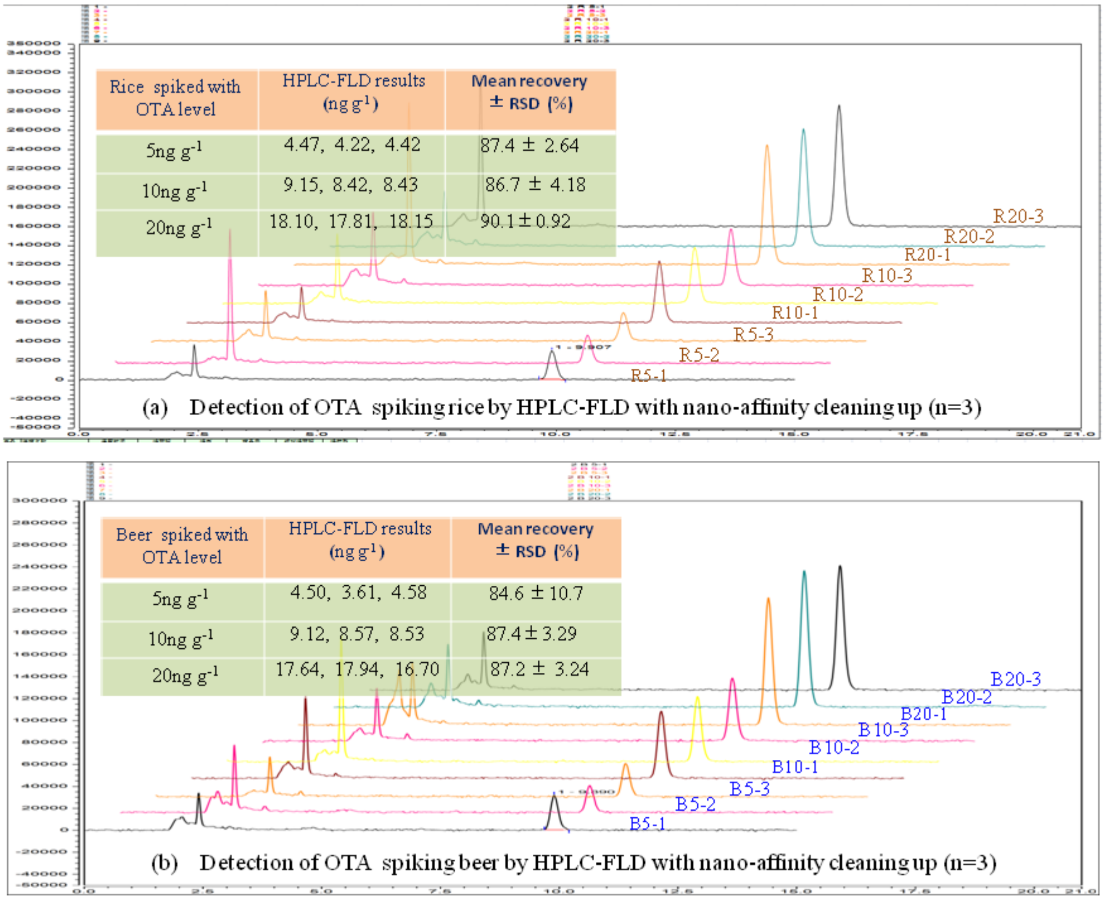

Figure S3. Detection of OTA spiking rice and beer samples by LC-FLD following nano-affinity cleaning up.
