# Supplementary material for: An Immuno-Separated Assay for Ochratoxin Detection Coupled with a Nano-Affinity Cleaning-Up for LC-Confirmation
Source: Foods. 2022 Apr 15;11(8):1155. doi: 10.3390/foods11081155 (PMC9026555; doi:10.3390/foods11081155)
Supplement: Supplementary file 1 [file foods-11-01155-s001.zip › Figure S4.pdf]

1. Detection of spiking sample by HPLC with nano-affinity cleaning up

To confirm the reliability of the immuno-separated assay, the collected IMAs should be eluted by 200μL 80% methanol solution (containing 0.5% acetic acid) for twice. The two elutes were combined and filtered by 0.1μm membrane for HPLC-FLD detection according to the reference (Campone et al. 2018).

1.1. Sample elution

(1) The collected IMAs were eluted with 200μL elution buffer by 10 mins shaking. The elution buffer were collected after separation.

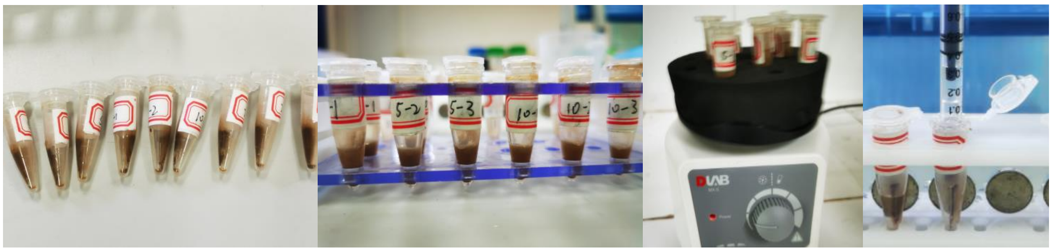

(2) Another elution, separation and collection.

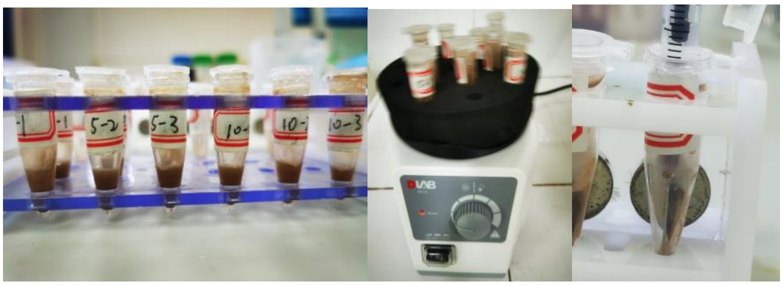

(3) Combine elutes for filter.

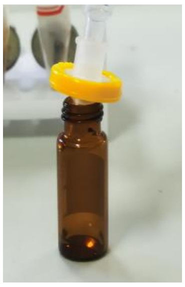

(4) Eluting solutions were detected by HPLC-FLD.

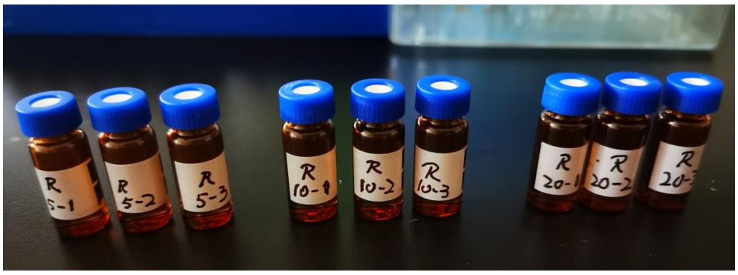

1.2. Detection results

(1) Detection results of rice samples by LC-FLD following nano-affinity cleaning up

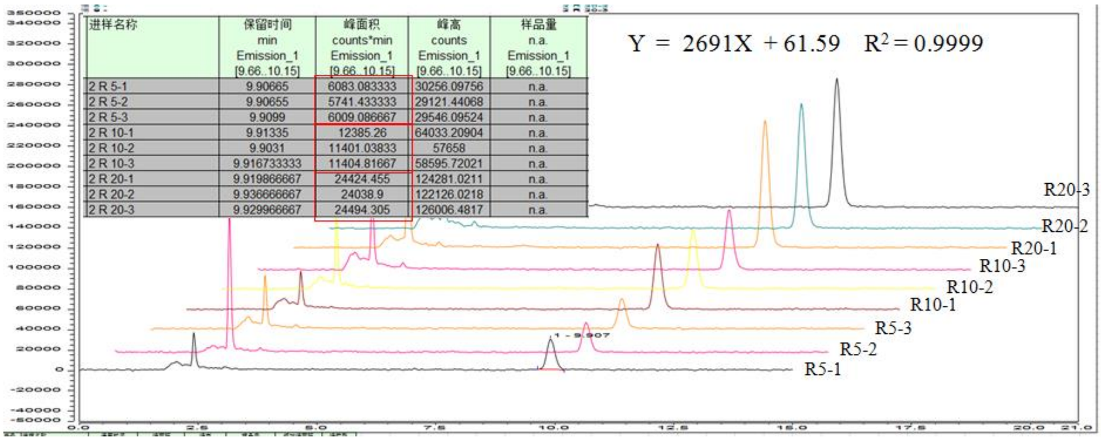

进样名称	保留时间 min Emission_1 [9.66..10.15]	峰面积 counts*min Emission_1 [9.66..10.15]	峰高 counts Emission_1 [9.66..10.15]	样品量 n.a. Emission_1 [9.66..10.15]
2 R 5-1	9.90665	6083.083333	30256.09756	n.a.
2 R 5-2	9.90655	5741.433333	29121.44068	n.a.
2 R 5-3	9.9099	6009.086667	29546.09524	n.a.
2 R 10-1	9.91335	12385.26	64033.20904	n.a.
2 R 10-2	9.9031	11401.03833	57658	n.a.
2 R 10-3	9.916733333	11404.81667	58595.72021	n.a.
2 R 20-1	9.919866667	24424.455	124281.0211	n.a.
2 R 20-2	9.936666667	24038.9	122126.0218	n.a.
2 R 20-3	9.929966667	24494.305	126006.4817	n.a.

(2) Detection results of beer samples by LC-FLD following nano-affinity cleaning up

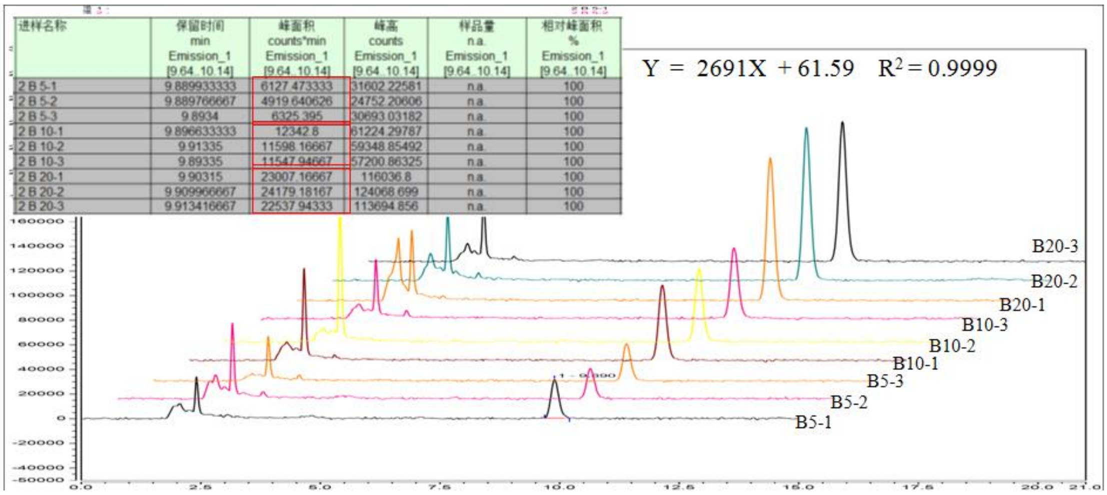

进样名称	保留时间 min Emission_1 [9.64..10.14]	峰面积 counts*min Emission_1 [9.64..10.14]	峰高 counts Emission_1 [9.64..10.14]	样品量 n.a. Emission_1 [9.64..10.14]	相对峰面积 % Emission_1 [9.64..10.14]
2 B 5-1	9.889933333	6127.473333	31602.22581	n.a.	100
2 B 5-2	9.889766667	4919.640626	24752.20606	n.a.	100
2 B 5-3	9.8934	6325.395	30693.03182	n.a.	100
2 B 10-1	9.896633333	12342.8	61224.29787	n.a.	100
2 B 10-2	9.91335	11598.16667	59348.85492	n.a.	100
2 B 10-3	9.89335	11547.94667	57200.86325	n.a.	100
2 B 20-1	9.90315	23007.16667	116036.8	n.a.	100
2 B 20-2	9.909966667	24179.18167	124068.699	n.a.	100
2 B 20-3	9.913416667	22537.94333	113694.856	n.a.	100

2. Detection of spiking sample by HPLC purified by immuno-affinity column

2.1. Commercial immuno-affinity column

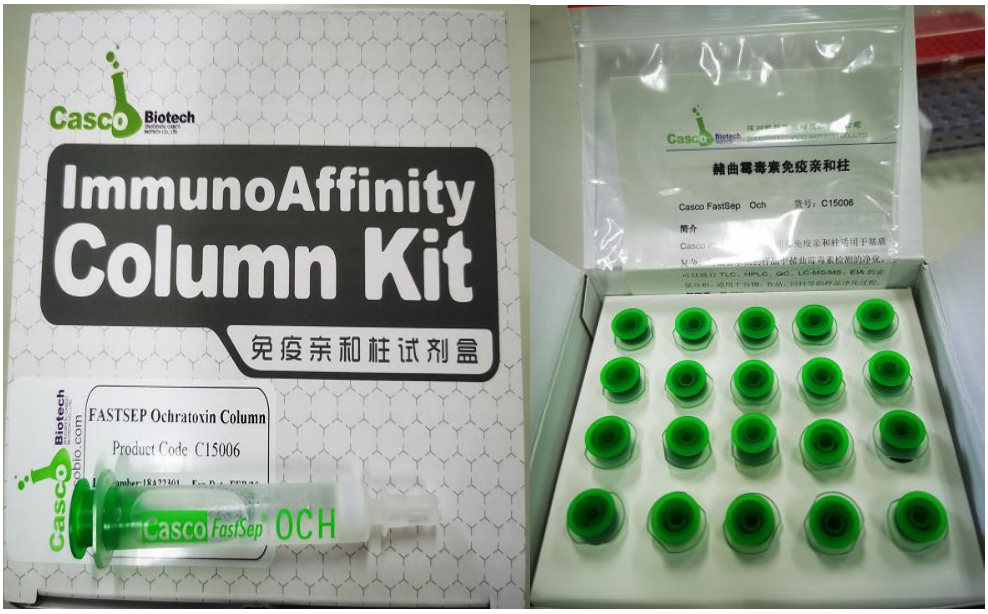

2.2. Sample elution

(1) 5.0g spiked rice samples were extracted by 10mL of 70% methanol.

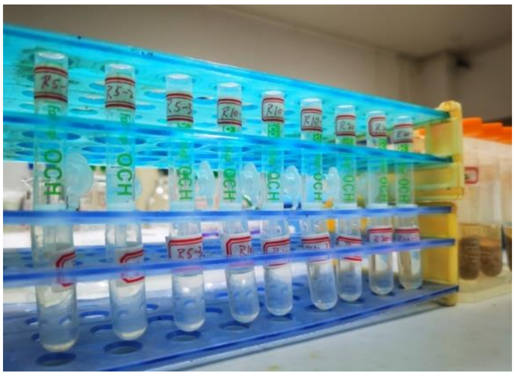

(2) 4.0mL extracting solutions were diluted with 0.01M PBS, then were loaded on the IAC.

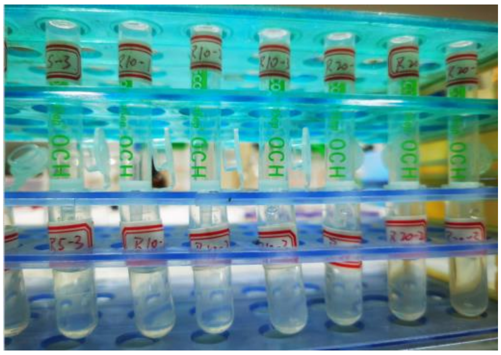

(3) The IAC were cleaned with 20mL 0.01M PBS and dried up. 2.0mL of 70% methanol (containing 0.5% formic acid) was used to eluted the IAC and collected the elutes.

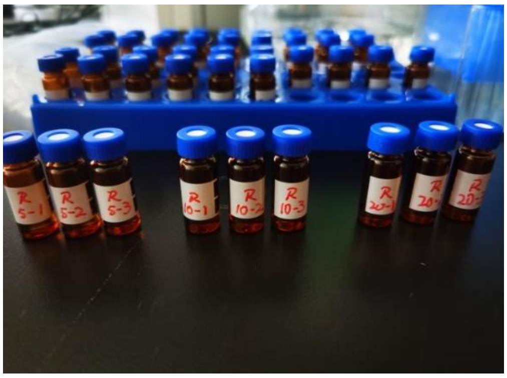

Rice samples

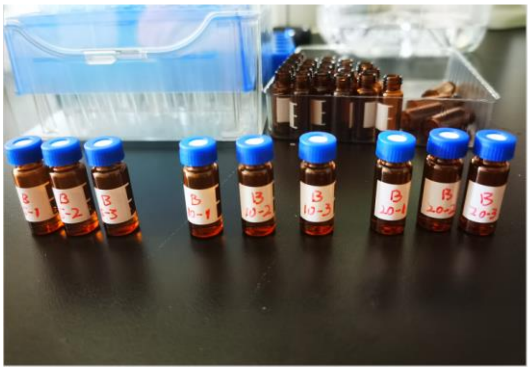

Beer samples

(4) The elutes were filtered for HPLC detection. The diluted ratios were 1:1.

2.3. *Detection results*

(1) Detection results of rice samples by LC-FLD following IAC cleaning up

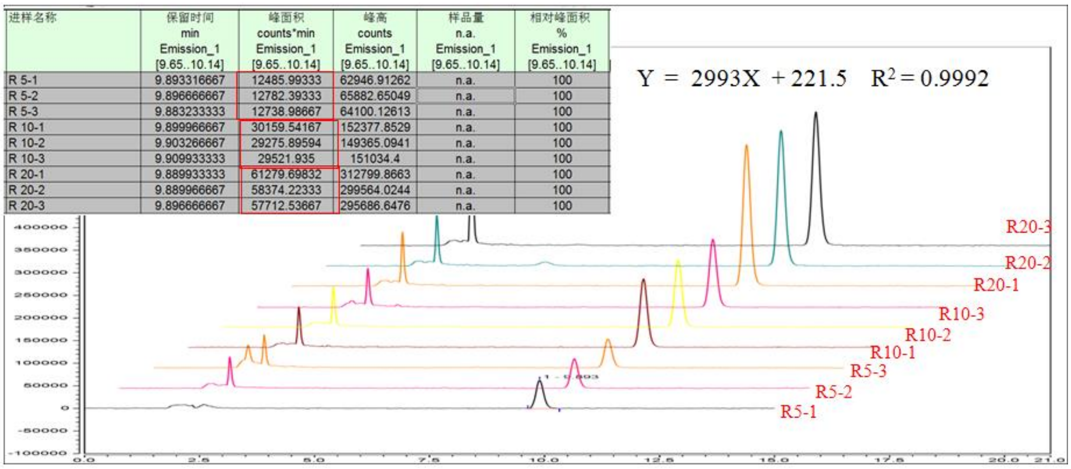

进样名称	保留时间 min Emission_1 [9.65..10.14]	峰面积 counts*min Emission_1 [9.65..10.14]	峰高 counts Emission_1 [9.65..10.14]	样品量 n.a. Emission_1 [9.65..10.14]	相对峰面积 % Emission_1 [9.65..10.14]
R 5-1	9.893316667	12485.99333	62946.91262	n.a.	100
R 5-2	9.896666667	12782.39333	65882.65049	n.a.	100
R 5-3	9.883233333	12738.98667	64100.12613	n.a.	100
R 10-1	9.899966667	30159.54167	152377.8529	n.a.	100
R 10-2	9.903266667	29275.89594	149365.0941	n.a.	100
R 10-3	9.909933333	29521.935	151034.4	n.a.	100
R 20-1	9.889933333	61279.69832	312799.8663	n.a.	100
R 20-2	9.889966667	58374.22333	299564.0244	n.a.	100
R 20-3	9.896666667	57712.53667	295686.6476	n.a.	100

(2) Detection results of beer samples by LC-FLD following IAC cleaning up

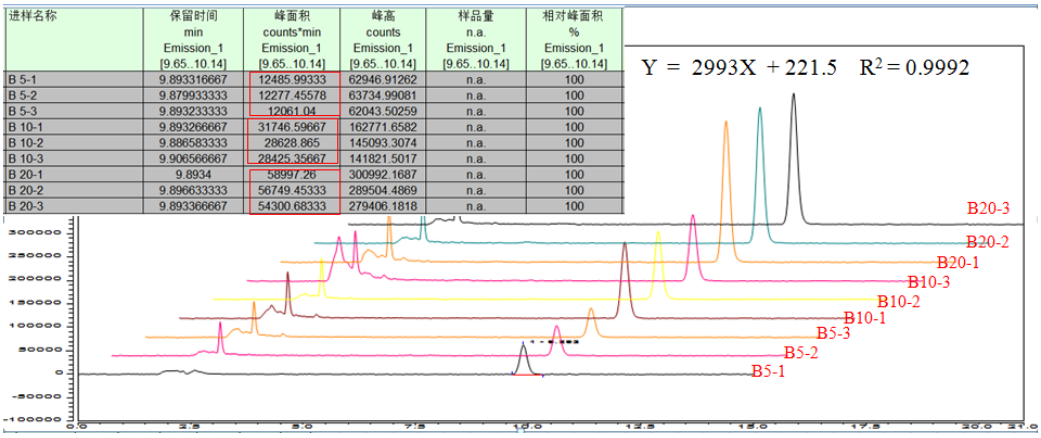

进样名称	保留时间 min Emission_1 [9.65..10.14]	峰面积 counts*min Emission_1 [9.65..10.14]	峰高 counts Emission_1 [9.65..10.14]	样品量 n.a. Emission_1 [9.65..10.14]	相对峰面积 % Emission_1 [9.65..10.14]
B 5-1	9.893316667	12485.99333	62946.91262	n.a.	100
B 5-2	9.879933333	12277.45578	63734.99081	n.a.	100
B 5-3	9.893233333	12061.04	62043.50259	n.a.	100
B 10-1	9.893266667	31746.59667	162771.6582	n.a.	100
B 10-2	9.886583333	28628.865	145093.3074	n.a.	100
B 10-3	9.906566667	28425.35667	141821.5017	n.a.	100
B 20-1	9.8934	58997.26	300992.1687	n.a.	100
B 20-2	9.896633333	56749.45333	289504.4869	n.a.	100
B 20-3	9.893366667	54300.68333	279406.1818	n.a.	100

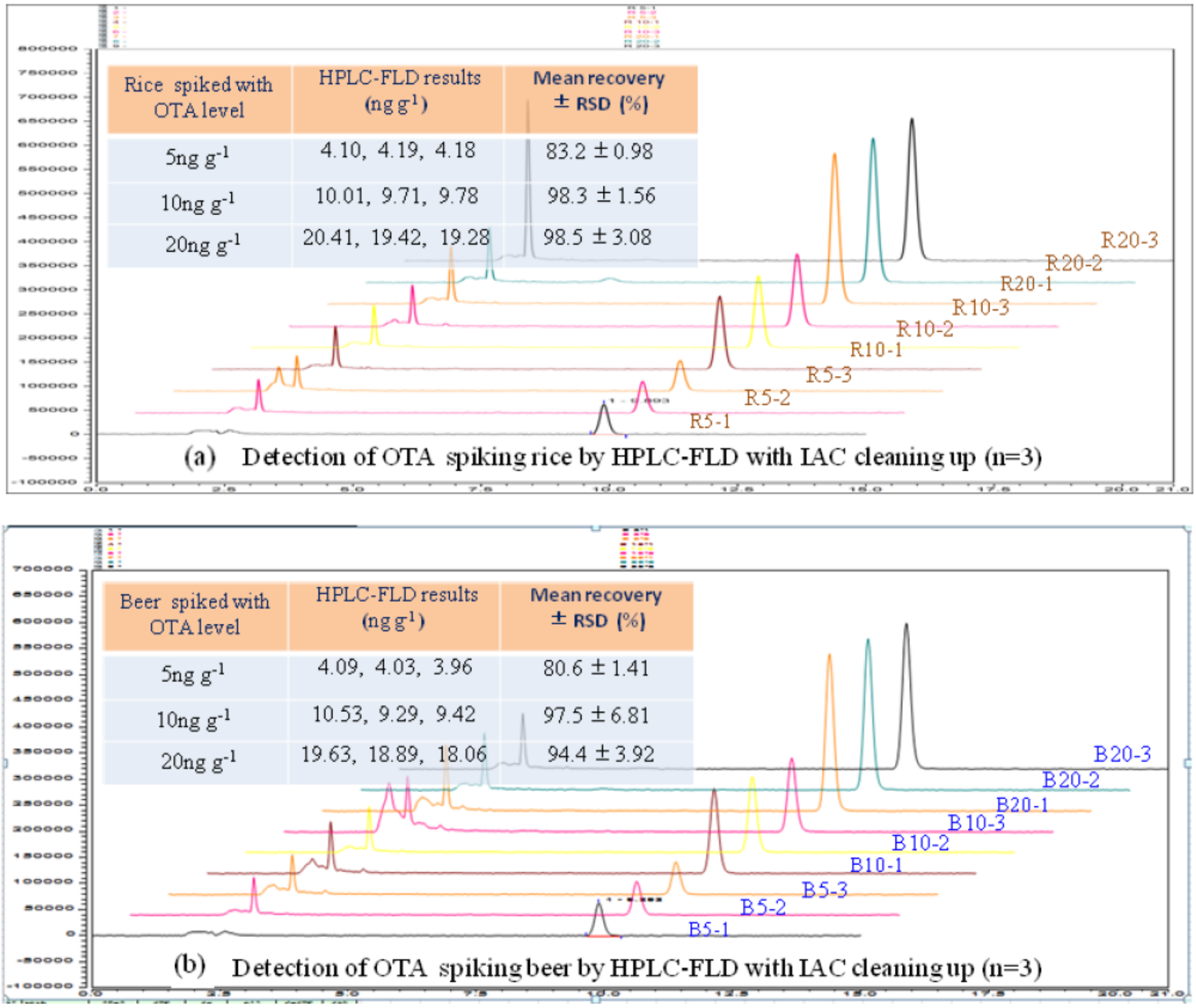

Figure S4. Detection of OTA spiking rice and beer samples by LC-FLD following immuno-affinity column cleaning up.
